# Asthma and health-related quality of life at 16–20 years of age in a prospectively followed post-bronchiolitis cohort

**DOI:** 10.1007/s00431-024-05754-6

**Published:** 2024-09-10

**Authors:** Sonja Laitinen, Eero Lauhkonen, Sanna Saarikallio, Riikka Riikonen, Ninni Keränen, Matti Korppi, Paula Heikkilä

**Affiliations:** 1 Tampere Center for Child, Adolescent and Maternal Health Research, Faculty of Medicine and Health Technology, Tampere University, Arvo Ylpön Katu 34, 33520 Tampere, Finland; 2 Department of Paediatrics, Tampere University Hospital, Wellbeing Services County of Pirkanmaa, P.O. Box 272, 33101 Tampere, Finland; 3 Department of Clinical Physiology and Nuclear Medicine, Tampere University Hospital, Wellbeing Services County of Pirkanmaa, P.O. Box 272, 33101 Tampere, Finland; 4 Division of Oncology, Surgery and Gastroenterology, Tampere University Hospital, Wellbeing Services County of Pirkanmaa, P.O. Box 272, 33101 Tampere, Finland

**Keywords:** Asthma, Bronchiolitis, Quality of life, Cohort study

## Abstract

The aim was to evaluate asthma and health-related quality of life (HRQoL) outcomes in adolescents, after hospital-treated bronchiolitis experienced in less than 6 months of age. A prospective cohort study started in 2001–2004 and followed up 166 children hospitalised for bronchiolitis in early infancy. At 16–20 years of age, 76 cases and 41 population-based controls without a history of bronchiolitis participated in the current study. Clinical asthma, presumptive symptoms and HRQoL data were collected with a structured questionnaire and the St. Georges Respiratory Questionnaire (SGRQ). Flow-volume spirometry was measured before and after bronchodilator administration. Asthma was present in 21.1% of cases in the post-bronchiolitis cohort compared to 9.8% in the control group (*p* = 0.21). Also, 35.5% of cases and 19.5% of controls reported dyspnea during the last 12 months (*p* = 0.04). In addition, total SGRQ scores were higher in the bronchiolitis group (4.26) than in the control group (1.67, *p* < 0.001) referring to a reduced health-related quality of life.

*Conclusion*: Viral bronchiolitis in early infancy was associated with increased respiratory symptoms and lower health-related quality of life at age 16–20 years.

**Table:** 

**What is known:** • The prevalence of asthma at the school age and adolescence is increased after hospitalisation required bronchiolitis in infancy compared to those without hospitalisation due to bronchiolitis.
**What is new:** • Viral bronchiolitis requiring hospitalisation in early infancy was associated with increased respiratory symptoms, such as dyspnoea, and lower health-related quality of life at age 16–20 years in a prospectively followed post-bronchiolitis cohort.

**What is known:**

• The prevalence of asthma at the school age and adolescence is increased after hospitalisation required bronchiolitis in infancy compared to those without hospitalisation due to bronchiolitis.

**What is new:**

• Viral bronchiolitis requiring hospitalisation in early infancy was associated with increased respiratory symptoms, such as dyspnoea, and lower health-related quality of life at age 16–20 years in a prospectively followed post-bronchiolitis cohort.

## Introduction

Bronchiolitis is the first wheezing episode associated with lower respiratory tract infection (LRTI) in infancy [1]. The upper age limit is 12 months in Europe and 24 months in America [2]. In birth cohort studies, 20–30% of children have experienced at least one parent-reported wheezing episode during the first 3 years of life, but less than 10% have needed hospitalisation [3].

Bronchiolitis is characterised by rhinitis, cough and tachypnoea, with wheezing or fine rales on auscultation [1, 2]. Respiratory syncytial virus (RSV) is the predominant causative pathogen before 12 months of age whereas rhinoviruses are the main causative agents in older infants [4]. Infants younger than 6 months are most likely to present with a severe disease course [1].

Bronchiolitis leading to hospitalisation in infancy is frequently associated with recurrent obstructive episodes and asthma in later life. The prevalence of post-bronchiolitis asthma has been 15–40% at school age and in adolescence in hospital-based post-bronchiolitis cohorts [5].

We have prospectively followed up until teenage initially 166 children who were hospitalised for bronchiolitis at younger than 6 months of age in 2000–2004 [6–9]. The aim of the present study was to investigate the presence of asthma, allergy, presumptive symptoms and health-related quality of life (HRQoL), at the age of 16–20 years in this post-bronchiolitis cohort, compared to population-based controls.

## Materials and methods

Originally, 187 infants were recruited in the study in 2001–2004, when they were hospitalised for bronchiolitis in the Department of Paediatrics, Tampere University Hospital. The inclusion criteria of the study were as follows: the infants had to be less than 6 months of age, previously healthy and born full term. Nasopharyngeal aspirates were collected from all children during hospitalisation, and the samples were screened for respiratory syncytial virus (RSV), influenza A and B virus, parainfluenza 1, 2 and 3 viruses, rhinoviruses, metapneumovirus and adenoviruses using antigen tests and polymerase chain reactions (PCR) [6].

### Previous control visits

At the age of 5–7 years, 166 children, and at the age of 11–13 years, 138 children participated in the follow-up visits [8, 9].

At both follow-up visits, a structured questionnaire was used to collect data on physician-diagnosed asthma and allergies of the child, self-reported symptoms, possible hospitalisations and annual and cumulative medications for asthma and allergy, as well as allergic diseases, asthma and smoking habits of the parents. Impulse oscillometry (IOS) and exercise challenge test with free running outdoors was done at 5–7 years and spirometry with bronchodilatation test at 11–13 years visits.

At the follow-up visit at 11–13 years, 664 controls matched for age and gender were randomly sampled from the national population register. Exclusion criteria for the control group were hospitalisation for any medical reason and outpatient treatment for bronchiolitis or any other LRTI during infancy. A total of 108 controls attended the clinic and performed the lung function testing.

### Current follow-up at 16–20 years of age

The current follow-up visits were organised from June 2021 to May 2022, when the study subjects were 16–20 years old. All 166 cases who attended the follow-up in 2008–2009 at the age of 5–7 years were invited. In addition, those 108 controls who participated in the previous follow-up at the age of 11–13 years were re-invited. The current visit consisted of clinical examination including measurement of height and weight, bronchodilatation test using flow-volume spirometry, a similar structured clinical questionnaire used previously in the study supplemented with questions about study subjects’ own tobacco smoking, and St. George’s Respiratory Questionnaire (SGRQ), which measures the HRQoL. The number of attending cases was 76 (45.8%): 72 attended the clinical visit and 4 returned the questionnaires only. The number of attending controls was 41 (40.0%): 29 attended the clinical visit and 13 returned the questionnaires only.

### Questionnaire data

Prior to the visit, the study subjects were sent a questionnaire, and the answers were revisited thoroughly with the investigator during the clinical visit. The questionnaire collected data regarding asthma diagnoses and symptoms, allergic rhinitis and other allergic diseases, wheezing, healthcare visits and hospitalisations for wheezing, and on family history of asthma and allergic diseases. Medications used to treat asthma, such as inhaled corticosteroids (ICS) and on-demand bronchodilators, were asked and registered by year since the follow-up visit at 11–13 years of age.

Smoking was evaluated by asking how many cigarettes the subjects had on average smoked per day during the last 12 months. Current smoking was defined as smoking at least one cigarette per day for the last year.

### Quality of life questionnaire

During the study visit, the subjects completed the SGRQ, which is a validated instrument designed to measure the impact on overall health, daily life and perceived well-being in patients with obstructive airway diseases [10]. The SGRQ results are expressed as symptom, activity and impact scores, summarised into total scores. Each score category ranges from 0 to 100 points, with higher scores indicating more limitations and lower HRQoL. The application was used to calculate the scores [11].

### Bronchodilation test

Flow-volume spirometry (Vyntus Pneumo, CareFusion, Germany) was studied before and 15 min after an inhalation of 0.3 mg salbutamol. Three technically acceptable pre- and post-inhalation curves were required, and the best pre-post values of forced expiratory volume in 1 s (FEV1) were used in the bronchodilation test. The test result was regarded as diagnostic for reversible bronchial obstruction, if FEV1 increased by at least 200 ml and 12% [12].

### Definition of asthma

Current asthma was defined as the presence of a previous asthma diagnosis together with regular use of ICSs as maintenance medication for asthma during the last 12 months. Furthermore, subjects who suffered from asthma-presumptive symptoms and in addition had a diagnostic increase in FEV1 in the bronchodilatation test were considered to have asthma. These symptoms included wheezing, prolonged (≥ 4 weeks) cough outside of infection, and chronic night cough.

### Definition of allergy

Current allergy was defined as seasonal or continuous symptoms of allergic rhinitis, allergic conjunctivitis or atopic dermatitis during the last 12 months. The study subjects had to have a diagnosis in question made by a doctor either during the last year or earlier. Allergic rhinoconjunctivitis was defined as the presence of either allergic rhinitis or conjunctivitis. Allergy was defined as the presence of allergic rhinitis or conjunctivitis or atopic dermatitis.

### Statistics

The data were managed with the SPSS 28.0 package (IBM, Armonk, NY). Univariate statistical analyses of categorised variables were studied with chi-square test, or Fisher’s exact test when appropriate, and those of continuous variables with *t*-test, if the distribution of the data was normal, or with Mann–Whitney *U* test, if the distribution was non-normal. Multivariate analyses were done with logistic regression, and age, sex, smoking and overweight (obesity included) were included as co-variates. Allergy was included as a co-variate in selected analyses. SGRQ scores were analysed as continuous variables and as categorised into two groups: under and over the median, as previously published [13]. Percentages, adjusted odds ratios (OR) and 95% confidence intervals (CI) were used to describe the data. The *p*-value of < 0.05 was considered statistically significant.

### Ethics

The study was approved by the Ethics Committee of the Tampere University Hospital District (R19070). Informed consent was obtained from all study subjects over 18 years old and from the parents before enrolling the children in infancy and during all control visits, including the current control visit, if the study subjects were under 18 years old.

## Results

The mean age of the 76 subjects was 18.1 years (range 16–20), and the number of boys was 39 (51.3%). The respective figures in 41 controls were 19.2 years (17–20) and 19 (46.3%). Since the groups were matched by age and sex, the differences were minor. Fifty-one (67.1%) cases had been positive for RSV and 5 (6.6%) for rhinovirus on admission.

Episodes of dyspnea during the last 12 months were more common in cases (35.5%) than in controls (19.9%; *p* = 0.04) (Table 1). About 10% of cases and controls reported use of ICSs and 17–20% use of bronchodilators, without any significant differences between the groups. Bronchodilation test was positive in 11.8% of cases and in none of controls (Table 1). Smoking was surprisingly common, 20–24%, as was overweight, 22–24%, without any significant differences between cases and controls (Table 1).

**Table 1:** Asthma-presumptive symptoms, medications and smoking for the last 12 months, and current weight status and positive bronchodilation test, in 76 cases in the post-bronchiolitis group and in 41 controls

	Bronchiolitis group (*n* = 76)	Control group (*n* = 41)	
*Finding*	*n (%)*	*n (%)*	*p-value*
Asthma-presumptive symptoms			
Dyspnea	27 (35.5)	7 (19.9)	**0.04**
Prolonged cough	8 (10.5)	2 (4.9)	0.30
Night cough	7 (9.2)	2 (4.9)	0.40
Medications for asthma			
Inhaled corticosteroids	8 (10.5)	4 (9.8)	0.90
Beta-sympathomimetics	13 (17.1)	7 (19.5)	0.75
Smoking	18 (23.7)	8 (19.5)	0.65
Weight status			
Obesity (BMI > 30)	9 (11.8)	3 (7.3)	0.45
Overweight* (BMI > 25)	18 (23.7)	9 (22.3)	0.86
Bronchodilation test			
FEV1 rise ≥ 12% and ≥ 200 ml	9 (11.8)	0 (0)	NC

^*^Overweight including obesity. *FEV1*, forced expiratory volume in 1 s. *NC*, not calculable

Asthma was present in 21.1% of 76 cases in the bronchiolitis group and in 9.8% of 41 controls (*p* = 0.21) (Table 2). Allergic rhinoconjunctivitis was more common (75.0%) in cases than in the controls (53.7%; *p* = 0.02) (Table 2).

**Table 2:** Asthma, allergic rhinitis and/or conjunctivitis and atopic dermatitis in 76 cases in the post-bronchiolitis group and in 41 controls

	Bronchiolitis group (*n* = 76)	Control group (*n* = 41)	
*Finding*	*n (%)*	*n (%)*	*p-value*
Asthma	16 (21.1)	4 (9.8)	0.21
Allergic rhinitis	51 (67.1)	20 (48.8)	0.08
Allergic conjunctivitis	37 (48.7)	13 (31.7)	0.08
Allergic rhinoconjunctivitis^1^	57 (75.0)	22 (53.7)	**0.02**
Atopic dermatitis	9 (11.8)	11 (26.8)	0.07
Allergy^2^	58 (76.3)	26 (63.4)	0.14

^1^Allergic rhinoconjunctivitis, either rhinitis or conjunctivitis

^2^Allergy, either allergic rhinoconjunctivitis or atopic dermatitis

The data of cases and controls was combined when the association of current risk factors with asthma outcomes was analysed. In univariate analyses, allergic rhinoconjunctivitis and allergy were statistically significant factors, whereas all other factors were non-significant (Table 3). In multivariate analyses, bronchiolitis in infancy increased the risk of asthma to 2.1-fold, when analyses were adjusted for age, sex, smoking and overweight, and to 1.7-fold, when further adjusted for allergic rhinoconjunctivitis (Table 4), although the results were not statistically significant.

**Table 3:** The current risk factors for asthma analysed as combined in 117 subjects of the bronchiolitis and control groups

	Asthma present (*n* = 20)	Asthma not present (*n* = 97)	
*Risk factor*	*n (%)*	*n (%)*	*p-value*
Sex (boys)	12 (60.0)	46 (47.4)	0.31
Smoking	5 (25.0)	21 (21.9) *	0.76
Overweight^1^	3 (15.0)	24 (25.3) **	0.33
Allergic rhinitis	15 (75.0)	56 (57.7)	0.16
Allergic rhinoconjunctivis^2^	18 (90.0)	61 (62.9)	**0.02**
Atopic dermatitis	6 (30.0)	14 (14.4)	0.09
Allergy^3^	18 (90)	66 (68)	**0.049**

^1^Overweight including obesity

^2^Allergic rhinoconjunctivitis, either rhinitis or conjunctivitis

^3^Allergy, either allergic rhinoconjunctivitis or atopic dermatitis

^*^Data lacking in 1 control; **data lacking in 2 controls

**Table 4:** The risk of asthma in 76 cases in the post-bronchiolitis group and in 41 controls in multivariate analyses

	Bronchiolitis group (*n* = 76)	Control group (*n* = 41)	*p-value*
Adjusted OR^1^ (95%CI)	2.14 (0.56–7.84)	1.0	0.25
Adjusted OR^2^ (95%CI)	1.71 (0.46–6.40)	1.0	0.43

*Adjusted OR*, adjusted odds ratio; *CI*, confidence interval. ^1^adjusted for age, sex, smoking and overweight; ^2^adjusted for age, sex, smoking, overweight and allergic rhinoconjunctivitis

The HRQoL assessed by the SGRQ scores was lower in cases than in controls, and the difference was statistically significant in all subcategories and in total scores as well (Table 5). In logistic regression, activity scores and total scores differed significantly and impact scores marginally between the groups (Table 6). Interestingly, current smoking was independently associated with impact scores (adjusted odds ratio 4.11; 95%CI 1.50–11.22).

**Table 5:** Quality of life assessed by the St. George`s Respiratory Questionnaire (SGRQ) scores comparing the bronchiolitis group with the control group in univariate analyses

	Bronchiolitis group (*n* = 76)	Control group (*n* = 41)	
*SGRQ components*	*Median (range) [IQ 25–75]*	*Median (range) [IQ 25–75]*	*p-value*
Symptom score	12.4 (0–59.4) [0–32.7]	10.2 (0–53.9) [0–15.0]	0.02
Activity score	5.96 (0–79.7) [0–19.9]	0.00 (0–49.0) [0.0–0.0]	< 0.001
Impact score	0.00 (0–49.8) [0–10.3]	0.00 (0–21.2) [0.0–0.0]	0.001
Total score	4.26 (0–55.6) [0–15.6]	1.67 (0–35.0) [0.0–3.6]	< 0.001

*IQ25–75*, interquartile range

**Table 6:** Quality of life assessed by the St. George`s Respiratory Questionnaire (SGRQ) scores comparing the bronchiolitis group with the control group in multivariate analyses

	Bronchiolitis group (*n* = 76)	Control group (*n* = 41)	
*SGRQ components*	Adjusted OR* (95% CI)	Adjusted OR* (95% CI)	*p-value*
Symptom score	2.00 (0.72–5.54)	1.00	0.185
Activity score	7.24 (2.08–25.2)	1.00	0.002
Impact score	3.26 (0.99–10.7)	1.00	0.052
Total score	9.22 (2.36–36.0)	1.00	0.001

*CI*, confidence interval; *OR*, odds ratio. *Adjusted for age, sex, current smoking and overweight

## Discussion

There are four main results in this prospectively followed post-bronchiolitis cohort study, which evaluated asthma and health-related quality of life at 16–20 years of age after hospitalisation due to bronchiolitis at < 6 months of age. First, asthma was rarer than expected, being present in 21.1% of former bronchiolitis patients compared to 9.8% of controls. The difference between cases and controls was not significant, which suggests, in addition to underpowered analyses, that there was a selection bias in controls. Second, 35.5% of cases and 19.9% of controls reported recent symptoms of dyspnea, which suggests that mild asthma without a confirmed asthma diagnosis was common, especially in former bronchiolitis patients. Third, asthma was often associated with allergic rhinoconjunctivitis, which is well-known in medical literature. In line, allergic rhinoconjunctivitis was more common in cases than in controls. Fourth, the HRQoL measured by the SGRQ instrument was lower at 16–20 years of age after infant bronchiolitis than in controls.

The prevalence of doctor-diagnosed asthma at 17–20 years of age after bronchiolitis was 30% in the Kuopio 1981–1982 cohort from Finland [14] and 43% in the Gothenburg 1984–1985 cohort from Sweden [15]. The respective figures were 64% after rhinovirus bronchiolitis and 43% after RSV bronchiolitis, compared to 12% in controls, in the Kuopio 1992–1993 cohort at the same age [16]. These three cohort studies were prospective but the age limit for bronchiolitis was 24 months, and one-fourth to one-fifth of asthma diagnoses were done by doctors at the control visits.

There are two long-term post-bronchiolitis cohorts, in which the age limit has been 12 months, and the follow-up has continued until 17–20 years of age. In a Swedish cohort including 46 subjects hospitalised for RSV bronchiolitis at age < 12 months and 92 controls, the prevalence of asthma and/or repeated wheezing in young adulthood was 39%, compared to 9% in controls [17]. In a Norwegian cohort with 225 young adults aged 17–20 years, who had been hospitalised for bronchiolitis in infancy at < 12 months of age, and with 167 matched population-based controls, current asthma was present in 25% in the post-bronchiolitis group compared to 13% in the control group [18]. In the Tucson birth cohort, the asthma prevalence after parent-reported early childhood wheezing treated at home was 20% at 16 years of age [3].

Our finding of 21.1% asthma prevalence was lower than in the cohorts from Finland and Sweden [14, 15], but close to the figures from Arizona, USA and Norway [3, 18]. Our diagnostic criteria were strict since half of asthma patients had used inhaled corticosteroids. The 35.5% figure of reporting episodes of dyspnea during the last 12 months suggests that there were subjects with mild asthma without asthma diagnosis, which has been commonly seen in adolescents and young adults [5].

Post-bronchiolitis asthma in this present cohort was lower than in previous studies for two reasons. First, the age limit of < 6 months means that the original group had a pure bronchiolitis in infancy without early-life asthma cases. Second, RSV was a common but rhinovirus a rare causative agent in infancy [8], and post-bronchiolitis asthma has been associated with a higher degree of rhinovirus bronchiolitis [4].

In European countries, like Finland, the incidence of allergic rhinitis in non-selected child populations has been 5–10% at the ages of 6–7 years and 23–31% at the ages of 13–14 years [19]. New sensitizations to inhaled allergens happen through school age leading to increasing symptoms. The prevalence of self-reported rhinoconjunctivitis was 14.3% at the age of 13–16 years in the population study from Umeå, Sweden [20]. These figures are much lower than those of the present study, where 67.1% of cases and 48.8% of controls reported allergic rhinoconjunctivitis.

Asthma was associated with allergic rhinitis and conjunctivitis. As many as 90% of study subjects with asthma reported allergic symptoms during the past year compared to only 62% of those without asthma. The association between asthma and allergic rhinitis and/or conjunctivitis is well-known in medical literature and needs to be taken into care in multivariate analyses [21]. Previously, Kuopio 1981–1982 cohort has documented the close connection between bronchial hyperreactivity, prolonged rhinitis and skin prick test positivity for inhaled allergens [13].

Health-related quality of life was reduced in the cohort consisting of previous bronchiolitis patients compared to the controls, and the result was confirmed for activity and total scores in multivariate analyses. The confidence intervals were large, speaking for underpowered analyses and/or for heterogeneous groups. The results are in line with previous observations from the Kuopio 1981–1982 cohort [13] and the Kuopio 1992–1993 cohort [22]. At the age of 28–31 years, former bronchiolitis and pneumonia patients had higher total SGRQ scores than controls, without any between-group differences in specific SGRQ domains [13]. At age 17–20 years, the SGRQ symptom scores were higher in former bronchiolitis patients; however, without significant between-group differences in total scores [23]. To our knowledge, other long-term post-bronchiolitis cohort studies have not evaluated quality of life. There is recent evidence that bronchiolitis has been associated with reduced quality of life in the short term after the disease in both children [24] and their parents [25].

The main limitation of the study was the small number of the cases and low attendance rate of the controls. The drop-out rate in the bronchiolitis group was 54%, which is, however, understandable since the prospective follow-up from infancy continued for longer than 15 years. Five years earlier, only 20% of invited controls attended the study, and since then, the dropout rate was as much as 63%. These figures mean that the analyses were underpowered and probably also biased, since symptomatic subjects are more willing to participate in medical studies than non-symptomatic ones. Despite these shortcomings, we were able to show that episodes of dyspnea were more common, and quality of life was lower in cases compared to controls.

The main strengths of the study were the prospective design and the long-term surveillance time. In addition, the material was unique, since due to the low age of < 6 months on admission, all patients had a pure bronchiolitis without any previous wheezing or other asthma symptoms. Since RSV caused over 70% of the cases, the results can be applied mainly to RSV bronchiolitis.

## Conclusion

This prospective long-term post-bronchiolitis follow-up cohort showed that episodes of dyspnea and reduced health-related quality of life were more common in former bronchiolitis patients than in controls at age 16–20 years.

## Data Availability

The anonymous data that support the findings of this study are available on the reasonable request from the corresponding author. The data are not publicly available due to privacy or ethical restrictions.

## Abbreviations

CI: Confidence intervals
FEV1: Forced expiratory volume in 1 s
HRQoL: Health-related quality of life
ICS: Inhaled corticosteroids
IOS: Impulse oscillometry
LRTI: Lower respiratory tract infection
OR: Odds ratios
PCR: Polymerase chain reactions
RSV: Respiratory syncytial virus
SGRQ: St. Georges Respiratory Questionnaire
